# Individual and Joint Associations of Cancer Diagnosis and Handgrip Strength with Depression in European Middle-Aged and Older Adults

**DOI:** 10.3390/cancers17050754

**Published:** 2025-02-23

**Authors:** Carlos Vasconcelos, Miguel Peralta, Adilson Marques

**Affiliations:** 1 Higher School of Education of Viseu, Ci&DEI, Polytechnic Institute of Viseu, 3504-510 Viseu, Portugal; 2 CIPER, Faculdade de Motricidade Humana, Universidade de Lisboa, 1495-751 Cruz Quebrada, Portugal; mperalta@fmh.ulsisboa.pt (M.P.); amarques@fmh.ulisboa.pt (A.M.); 3 ISAMB, Faculdade de Medicina, Universidade de Lisboa, 1649-028 Lisboa, Portugal

**Keywords:** risk factors, cancer, muscle strength, mental health, interaction, aged, Europe

## Abstract

**Background/Objectives:** Depression is influenced by a multitude of interconnected factors, including cancer and handgrip strength. The purpose of our study is to examine how handgrip strength and cancer diagnosis are individually and jointly associated with depression. **Methods:** An observational study was employed using secondary data from the Survey of Health, Aging, and Retirement in Europe waves 7 and 8. The sample comprised 7 641 participants (71.1 ± 7.7 years), from 12 European countries. **Results:** Having a cancer diagnosis was associated with greater odds of depression (depression 2017 model: OR = 1.35, 95% CI = 1.03, 1.75; depression 2019 model: OR = 1.48, 95% CI = 1.12, 1.95). Being in a higher handgrip tertile was associated with lower odds of depression (depression 2017 model: tertile 2, OR = 0.65, 95% CI = 0.56, 0.74 and tertile 3, OR = 0.56, 95% CI = 0.49, 0.65; depression 2019 model: tertile 2, OR = 0.74, 95% CI = 0.64, 0.86 and tertile 3, OR = 0.76, 95% CI = 0.65, 0.88). The greatest reduction in odds for depression was found for participants without cancer and in the third handgrip strength tertile (depression 2017: OR = 0.42, 95% CI = 0.29, 0.61; depression 2019: OR = 0.40, 95% CI = 0.28, 0.58). **Conclusions:** Both cancer and handgrip strength were associated with depression, with the first increasing its risk and the latter reducing it. Individuals without cancer and in the highest tertile of handgrip strength have lower odds of depression in both analyses.

## Simple Summary

Depression is a mood disorder affecting millions of people worldwide and is especially prevalent in older ages. There are a multitude of interconnected factors that influence depression, including cancer and handgrip strength. In this study, we sought to understand how these two factors, cancer diagnosis and handgrip strength, are individually and jointly associated with depression. For that, we conducted an analysis with depression evaluated in 2017 and 2019 in a large sample of European middle-aged and older adults. Both cancer and handgrip strength were associated with depression, with the first increasing its risk and the later reducing it. A joint association pattern was also found, where individuals with greater handgrip strength and no cancer had a lower risk of depression. Campaigns aimed at cancer prevention and participation in exercise programs, with an emphasis on resistance training, should be encouraged.

## 1. Introduction

Depression is a mental health disorder that ranks as the 14th leading cause of disability-adjusted life years among individuals aged 50 to 74 years [1] and is associated with an increased risk of all-cause and cardiovascular mortality in this population [2,3]. The prevalence of depressive symptoms among middle-aged and older adults is alarming. It is estimated that 31.7% of individuals aged 45 and older experience such symptoms [4]. According to Remes et al. [5], depression is influenced by a multitude of interconnected factors, including cancer and handgrip strength.

Cancer is the leading cause of premature death in most European countries [6]. The global cancer burden is projected to reach 28.4 million cases by 2040, a 47% increase from 2020 [7]. The likelihood of depression among cancer patients is more than five times greater than that of the general population [8], leading to unfavorable outcomes such as low adherence to medical treatment [9], shorter survival rates [10], and an elevated risk of suicide [11].

Handgrip strength is a simple yet effective measure of overall muscle strength and functional capacity standing as an important biomarker of health [12]. Several studies have demonstrated an inverse relationship between handgrip strength and depression among middle-aged and older adults [13,14,15]. This suggests that individuals with lower handgrip strength may face physical limitations and increased psychological implications [16], ultimately worsening their overall health [17].

The individual associations between cancer and handgrip strength with depression are well established, with one being a risk factor and the other a protective factor. However, the interaction between these two, cancer and handgrip strength, and their joint effect on the risk of depression is still understudied. Furthermore, to the best of our knowledge, there is a lack of large European multicountry studies investigating the individual and combined associations between handgrip strength and cancer with depression in middle-aged and older adults. Understanding this relationship is crucial, as it may help identify subgroups at a higher risk for depression, allowing for more targeted interventions. Thus, the present investigation aims to examine how handgrip strength and having a cancer diagnosis are individually and jointly associated with depression (evaluated in two different moments) in a large sample of European middle-aged and older adults.

## 2. Materials and Methods

### 2.1. Study Design and Procedures

An observational analytical study design was employed using secondary data from the Survey of Health, Aging, and Retirement in Europe (SHARE) waves 7 and 8. SHARE is a biannual survey with physical examination that collects data on individuals aged 50 and over in several European countries and Israel, providing a multidisciplinary view to the challenges of population aging. Data were collected between March and November 2017 for wave 7 and from October 2019 to March 2020, before the COVID-19 pandemic, for wave 8.

In SHARE, data are collected through face-to-face interviews, about 90 min long, at the participant’s residence using Computer-Assisted Personal Interview. In each country, data collection was the responsibility of a national organization following the same approach. More details are available at the SHARE methodology volumes (https://share-eric.eu/data/data-documentation/methodology-volumes, accessed on 16 November 2024). SHARE protocol was approved by the Ethics Council of the Max-Planck Society for the Advancement of Science, verifying the procedures to guarantee confidentiality and data privacy.

### 2.2. Participants

For this investigation, the study population consisted of European and older adults (50 years and older). Adults and older adults participating in both waves 7 and 8 of SHARE and with valid data on cancer diagnosis, handgrip strength, depression symptoms, age, sex, country, education level, and multimorbidity were included in the study. Thus, the sample comprised 7 641 participants, mean age ± standard deviation (SD) = 71.1 ± 7.7 years, from 12 European countries (Austria, Germany, Sweden, Spain, Italy, France, Denmark, Greece, Switzerland, Belgium, Czech Republic, and Poland).

### 2.3. Measures

Two exposures were assessed in wave 7 (baseline): cancer diagnosis and handgrip strength. The cancer diagnosis was self-reported. Participants were asked whether a medical doctor had informed them that they had cancer. Participants who responded affirmatively to this question were considered to have a cancer diagnosis. Handgrip strength was measured using a handheld dynamometer (Smedley, S Dynamometer, TTM, Tokyo, Japan, 100 kg). Participants performed the test either standing or sitting with their elbow fixed at a 90° angle and a neutral wrist position, being instructed to squeeze the dynamometer with each of their hands as hard as possible and maintain it for 5 s. The highest grip strength value attained is recorded. The handgrip variable was considered valid only for participants with at least two measures and if the two measures did not differ >20 kg. As handgrip significantly differs between sexes and declines with age [18,19], sex- and age-specific tertiles were calculated. Higher tertiles indicate stronger grip strength for a particular sex and age group.

Depression symptoms, the outcome, were measured in wave 7 and wave 8 using the EURO-D 12-item score [20]. The scores range between 0 and 12, with higher scores indicative of higher depressive symptomatology and a cut-off point of 4 or more points greatly correlated with clinically significant depression.

A set of covariates was also self-reported, including sex, age, country, education level, and multimorbidity. The International Standard Classification of Education (ISCED) codes assessed the education level. To assess multimorbidity, participants were asked to report the presence or absence of several diseases diagnosed by a medical doctor. The number of chronic diseases was summed to produce a score and classify participants as having or not multimorbidity (≥2 diseases).

### 2.4. Data Analysis

Descriptive statistics, including mean, SD, and frequencies, were calculated for all variables. Multivariable binary logistic regression models were conducted to assess the individual and joint association between cancer diagnosis and handgrip strength with depression evaluated in 2017 and depression evaluated in 2019. Two models, unadjusted (model 1) and adjusted (model 2), were performed. Model 2 was adjusted for sex, age, country, educational level, and multimorbidity in the analysis where depression was evaluated in 2017 and further adjusted for baseline depression symptoms in the analysis where depression was evaluated in 2019. For the individual analysis, cancer diagnosis and sex- and age-specific handgrip tertiles entered singularly as predictors of depression. Not having a cancer diagnosis and being in the 1st tertile (lowest) of the handgrip were set as the reference groups. A new variable was created for the joint analysis by combining the cancer diagnosis and handgrip tertiles groups. The new variable had 6 groups: (1) cancer diagnosis and handgrip in tertile 1, (2) cancer diagnosis and handgrip in tertile 2, (3) cancer diagnosis and handgrip in tertile 3, (4) no cancer diagnosis and handgrip in tertile 1, (5) no cancer diagnosis and handgrip in tertile 2, and (6) no cancer diagnosis and handgrip in tertile 3. The first group of this new variable (cancer diagnosis and handgrip in tertile 1) was set as the reference group for the joint analysis. All data analysis was performed using SPSS Statistics for Windows, version 25 (IBM Corp, Armonk, NY, USA). Statistical significance was set at *p* < 0.05.

## 3. Results

Participants were 3274 (42.8%) men and 4367 (57.2%) women with 71.1 ± 7.7 years of mean age in 2017 from 12 European countries. At baseline, most participants had multimorbidity (4173, 54.6%), almost a quarter had a EURO-D score equal to or higher than 4 (1772, 23.2%), and 301 (3.9%) reported a cancer diagnosis. More details about the participants’ characteristics are presented in Table 1. The percentages of participants in each handgrip tertile by cancer status in 2017 are shown in Figure S1 (Supplementary Materials).

Table 2 shows individual associations between cancer and handgrip strength evaluated in 2017 with depression evaluated in 2017, while Table 3 shows that associations with depression evaluated in 2019. In the unadjusted and adjusted models, having a cancer diagnosis was associated with greater odds of depression (depression in 2017 model 2: OR = 1.35, 95% CI = 1.03, 1.75; depression in 2019 model 2: OR = 1.48, 95% CI = 1.12, 1.95). On the other hand, being in a higher handgrip tertile was associated with a 35% (tertile 2, 95% CI = 0.56, 0.74) and 44% (tertile 3, 95% CI = 0.49, 0.65) decrease in the odds of depression evaluated in 2017 and a 26% (tertile 2, 95% CI = 0.64, 0.86) and 24% (tertile 3, 95% CI = 0.65, 0.88) decrease in the odds of depression evaluated in 2019.

**Figure 1:**
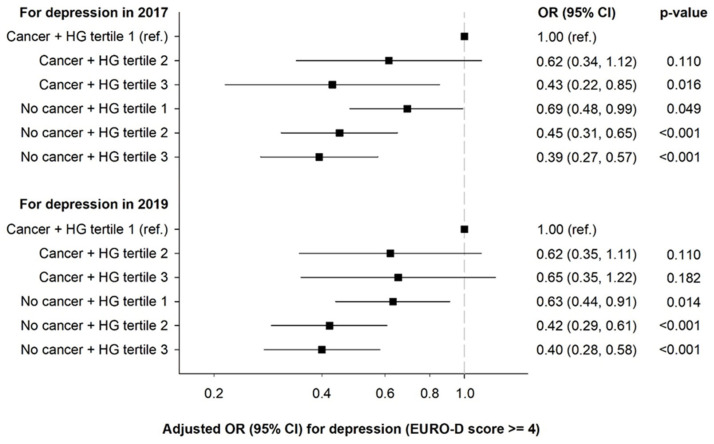
Joint associations between cancer and handgrip strength in 2017 with depression in 2017 and 2019. Analyses were adjusted for sex, age, country, education level, multimorbidity, and depression symptoms (only for analysis of depression in 2019). Abbreviations: CI, confidence interval; HG, handgrip; OR, odds ratio.

The joint association analyses showed a protective effect of better relative handgrip strength on depression both in the presence and absence of a cancer diagnosis (see Figure 1). The analysis where depression was evaluated in 2017 revealed that those with a cancer diagnosis and in the highest handgrip tertile had 57% lower odds of depression (OR = 0.43, 95% CI = 0.22, 0.85) similar to those without a cancer diagnosis, as compared to participants with cancer and in the lower handgrip strength tertile. The same trend was observed in the analysis where depression was evaluated in 2019 but statistically non-significant. In both models, the greatest reduction in odds for depression (>50%) was found for participants without cancer and in the second (depression evaluated in 2017: OR = 0.45, 95% CI = 0.31, 0.65; depression evaluated in 2019: OR = 0.39, 95% CI = 0.27, 0.57) and third (depression evaluated in 2017: OR = 0.42, 95% CI = 0.29, 0.61; depression evaluated in 2019: OR = 0.40, 95% CI = 0.28, 0.58) handgrip tertiles.

## 4. Discussion

The present study examined the individual and joint associations of having a cancer diagnosis and handgrip strength with depression in European middle-aged and older adults. Our findings revealed that having a cancer diagnosis was associated with greater odds of depression, while being in a higher handgrip tertile was linked with lower odds of depression in both analyses. Furthermore, in the joint association analysis, a protective effect of better relative handgrip strength on depression both in the presence and absence of a cancer diagnosis was observed.

Previous research has consistently associated cancer diagnosis with greater depression symptomatology. It seems that cancer patients experienced depression more often compared to healthy individuals (23.9% vs. 16.6%) [21]. Cancer patients are five times more likely to have depression than people without cancer. However, the likelihood of depression varied by cancer type, with prostate cancer having the lowest odds and brain cancer the highest [8]. The link between cancer and depression can be attributed to several interrelated risk factors, which can be grouped into four domains: cancer-specific, biological, social, and psychological [22]. Some risk factors differ at each stage of the cancer journey, from the moment of diagnosis, through treatment, to the phase of survivorship. Among other examples, the type of cancer is a cancer-specific factor relevant in the diagnosis phase. Comorbidities and lack of health insurance, which represent biological and social factors, respectively, are significant during the treatment phase. Finally, fear of cancer recurrence, a psychological concern, arises during the survivorship phase [22]. The presence of several risk factors for depression across the different phases of the cancer process supports the findings of our study, which show a significantly higher risk of depression among cancer patients, in both analyses.

Handgrip strength is an indicator of health and vulnerability [23], with low grip strength inversely associated with a variety of health outcomes, including multimorbidity [24], functional disabilities [25], and mental health issues, such as depression [23,26]. Similarly to our findings, recent research has highlighted that lower handgrip strength increases the odds of depression among middle-aged and older adults, both cross sectionally and longitudinally. Results have shown that having lower handgrip strength doubled the odds of experiencing depressive symptoms [14]. Likewise, longitudinal studies also show that lower baseline handgrip strength was associated with developing depression, with assessments conducted 1 year [27] and 4 years later [28]. Several neurobiological mechanisms were proposed to explain the link between handgrip and depression. To begin with, decreased muscle strength was associated with elevated levels of inflammatory markers in older adults [29], which are closely related to the core mechanisms of depression [30]. Additionally, as a simple yet powerful measure of muscular strength [31], handgrip is positively associated with moderate-to-vigorous physical activity [32]. Physical activity levels can influence the production and regulation of neurotransmitters such as dopamine, which play a pivotal role in the modulation of depressive symptoms [33]. Moreover, improved physical health is linked to the increased release of brain-derived neurotrophic factor (BDNF) [34], a contraction-induced myokine that is inversely associated with depression [35]. Furthermore, research has demonstrated that higher muscle strength is associated with increased gray matter volume in brain regions linked to emotional regulation and cognitive function [36]. Conversely, deficits in gray matter volume may play a significant role in the etiology of depression [37]. Psychosocial aspects may also justify the association between handgrip strength and depression. Impaired physical functioning, which leads to disabilities in activities of daily living [38], can affect feelings of achievement [39], self-efficacy, and self-esteem [40], three protective factors for depression symptoms. Furthermore, middle-aged and older adults with greater mobility and less disability are more likely to participate in social activities and receive social support, which can contribute to a reduced risk for depression [41].

Cancer and handgrip strength are both individually associated with depression. To the best of our knowledge, we assessed for the first time their joint association. Firstly, the protective effect of not having cancer on depression was evident even in the people with lower handgrip strength, as observed in both the analysis where depression was evaluated in 2017 (31% reduction in odds) and the analysis where depression was evaluated in 2019 (37% reduction in odds). The aforementioned risk factors for depression in cancer patients may explain this finding. Secondly, a protective effect of better relative handgrip strength was also found, mainly in people without cancer. Among people with cancer, the protective effect of handgrip was less evident. Only in the analysis where depression was evaluated in 2017 was a significant reduction in odds observed for those in the highest handgrip tertile. However, all the other groups presented a protective trend for better handgrip strength. These results should be interpreted with caution because of the low number of participants with cancer. In addition, some confounding factors that emerge between both assessments can influence these non-significant results. Individuals with cancer frequently experience a decrease in physical activity due to treatment-related fatigue and physical limitations [42,43], which can adversely affect both handgrip strength and mental health. Another possible explanation is related to the decline of handgrip strength over time, which may be influenced by cancer progression and its treatment side effects [44]. These factors, which are not captured in a single cross sectional measurement (2017), can significantly impact the relationship between handgrip strength and depression in cancer patients in the analysis where depression was evaluated in 2019. Notwithstanding, the inverse association between handgrip strength and depression was also observed in investigations comparing groups of cancer patients. In a recent study, involving 876 cancer survivors (mean age of 64.7 ± 13.8 years), it was concluded that having low handgrip strength increased two-fold the risk of depression compared to those with normal handgrip strength (OR = 2.02; 95% CI = 1.07, 3.81) [45]. Therefore, our findings, supported by previous evidence, suggest that having better handgrip strength may protect against depression even in the presence of a cancer diagnosis.

The present study has some limitations that should be considered when interpreting the results. The use of a self-reported questionnaire to assess depression may introduce bias, as the prevalence of this condition could be overestimated. This limitation could affect the accuracy of the collected data and, consequently, the interpretation of the observed associations. In addition, reduced sample sizes in the groups with cancer and some confounding factors that may arise from one analysis (depression evaluated in 2017) to another (depression evaluated in 2019) may have limited the statistical power to detect significant differences. Another limitation is that our analysis does not allow for the determination of causality. We also acknowledge that without an interaction analysis, we cannot fully capture how the relationship between grip strength and depression varies based on cancer status. Despite these limitations, this study presents several strengths that enhance the validity of its results. Firstly, the creation of the handgrip strength tertiles was stratified by sex and age, which allows for a more nuanced understanding of the data. Additionally, adjusting for various covariates provides deeper insights into the interactions between the variables. Furthermore, the two-year follow-up period demonstrates a temporal association, offering more robust evidence regarding the relationship between the variables studied. Another strength of the present study is the use of a large and representative sample from several European countries. This geographical and cultural diversity increases the generalizability of the results, allowing the conclusions to be applicable to a broader population.

## 5. Conclusions

Both cancer and handgrip strength were associated with depression, with the first increasing its risk and the latter reducing it. Furthermore, the joint association allowed for the observation that individuals without cancer and in the highest tertile of handgrip strength have lower odds of depression in both analyses. From a public health perspective, these findings emphasize the importance of improving muscular strength to counteract the detrimental effects of cancer and low handgrip strength on depression. Campaigns aimed at cancer prevention and participation in exercise programs, with an emphasis on resistance training, should be encouraged. Future studies should incorporate an interaction analysis of cancer diagnosis and handgrip strength on depression. In addition, they should aim to include the type of cancer and kind of therapy to better understand the studied associations.

## Figures and Tables

**Table 1:** Participants’ characteristics.

	Mean ± SD or *n* (%)
**Sex**	
Men	3274 (42.8)
Women	4367 (57.2)
**Age (years)**	71.1 ± 7.7
**Country**	
Austria	184 (2.4)
Germany	573 (7.5)
Sweden	691 (9.0)
Spain	559 (7.3)
Italy	689 (9.0)
France	752 (9.8)
Denmark	819 (10.7)
Greece	1086 (14.2)
Switzerland	557 (7.3)
Belgium	613 (8.0)
Czech Republic	584 (7.6)
Poland	534 (7.0)
**Education level**	
None	226 (3.0)
ISCED 1	1788 (23.4)
ISCED 2	1145 (15.0)
ISCED 3	2346 (30.7)
ISCED 4	356 (4.7)
ISCED 5	1702 (22.3)
ISCED 6	78 (1.0)
**Multimorbidity**	
Yes	4173 (54.6)
**Depression symptoms 2017 (EURO-D score)**	2.2 ± 2.1
**Depression 2017 (EURO-D score ≥ 4)**	
Yes	1772 (23.2)
**Depression symptoms 2019 (EURO-D score)**	2.3 ± 2.2
**Depression 2019 (EURO-D score ≥ 4)**	
Yes	1925 (25.2)
**Cancer**	
Yes	301 (3.9)
**Handgrip strength (kg)**	31.9 ± 10.9
**Cancer and handgrip groups**	
Cancer + handgrip tertile 1	136 (1.8)
Cancer + handgrip tertile 2	92 (1.2)
Cancer + handgrip tertile 3	73 (1.0)
No cancer + handgrip tertile 1	2745 (35.9)
No cancer + handgrip tertile 2	2321 (30.4)
No cancer + handgrip tertile 3	2274 (29.8)

ISCED, International Standard Classification of Education.

**Table 2:** Individual associations between cancer and handgrip strength evaluated in 2017 with depression evaluated in 2017.

	Depression (EURO-D Score ≥ 4) in 2017			
	Model 1 OR (95%CI)	*p*-Value	Model 2 OR (95%CI)	*p*-Value
Cancer				
No	1.00 (ref)		1.00 (ref)	
Yes	1.51 (1.17, 1.94)	0.001	1.35 (1.03, 1.75)	0.028
Handgrip strength				
Tertile 1	1.00 (ref)		1.00 (ref)	
Tertile 2	0.58 (0.51, 0.66)	<0.001	0.65 (0.56, 0.74)	<0.001
Tertile 3	0.47 (0.41, 0.54)	<0.001	0.56 (0.49, 0.65)	<0.001

Model 1: Unadjusted; Model 2: Adjusted for sex, age, country, education level, multimorbidity. Abbreviations: CI, confidence interval; OR, odds ratio.

**Table 3:** Individual associations between cancer and handgrip strength evaluated in 2017 with depression evaluated in 2019.

	Depression (EURO-D Score ≥ 4) in 2019			
	Model 1 OR (95%CI)	*p*-Value	Model 2 OR (95%CI)	*p*-Value
Cancer				
No	1.00 (ref)		1.00 (ref)	
Yes	1.74 (1.37, 2.22)	<0.001	1.48 (1.12, 1.95)	0.006
Handgrip strength				
Tertile 1	1.00 (ref)		1.00 (ref)	
Tertile 2	0.60 (0.53, 0.68)	<0.001	0.74 (0.64, 0.86)	<0.001
Tertile 3	0.54 (0.48, 0.62)	<0.001	0.76 (0.65, 0.88)	<0.001

Model 1: Unadjusted; Model 2: Adjusted for sex, age, country, education level, multimorbidity, and depression symptoms. Abbreviations: CI, confidence interval; OR, odds ratio.

## Data Availability

The original data presented in the study are openly available in the SHARE research data center at https://share-eric.eu/data/, accessed on 16 November 2024.

## Supplementary Materials

The following supporting information can be downloaded at: https://www.mdpi.com/article/10.3390/cancers17050754/s1, Figure S1: Percentage of participants in each handgrip tertile by cancer status in 2017.
