# Supplementary material for: Individual and Joint Associations of Cancer Diagnosis and Handgrip Strength with Depression in European Middle-Aged and Older Adults
Source: Cancers (Basel). 2025 Feb 23;17(5):754. doi: 10.3390/cancers17050754 (PMC11899050; doi:10.3390/cancers17050754)
Supplement: Supplementary file 1 [file cancers-17-00754-s001.zip › cancers-3396815-supplementary.pdf]

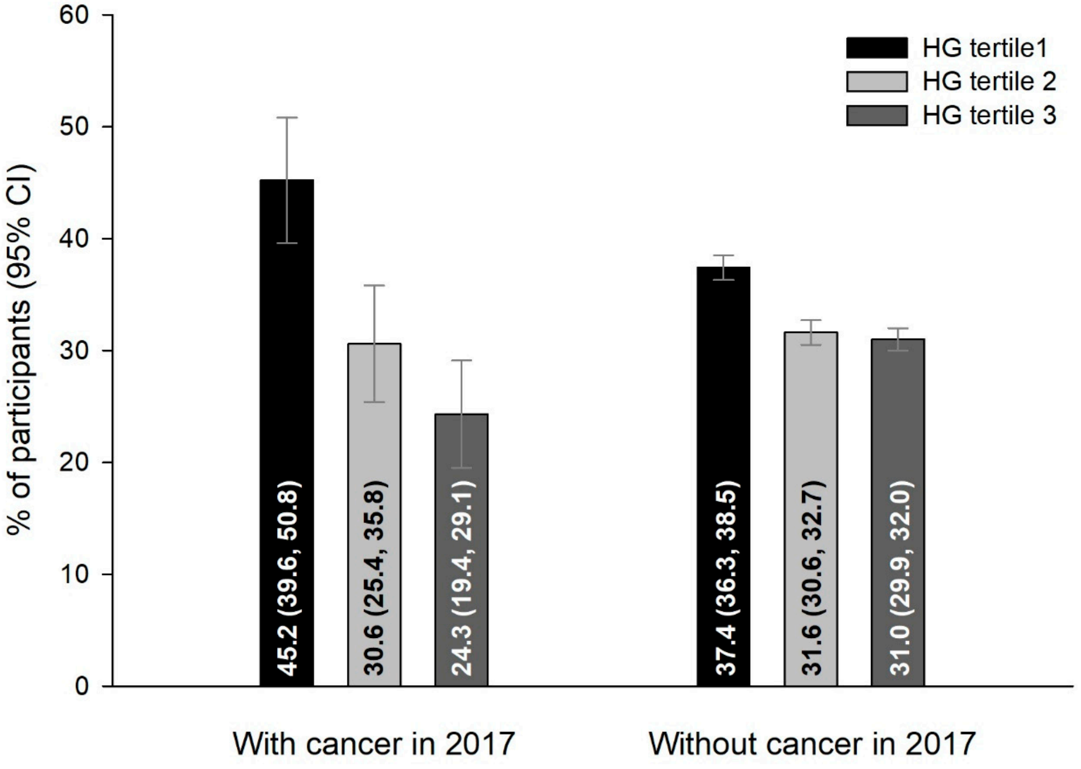

Figure S1. Percentage of participants in each handgrip tertile by cancer status in 2017.

Abbreviations: CI, confidence interval; HG, handgrip.
